# Imaging-Based Parallel Seismic Test for In-Service Bridge Pile Foundations

**DOI:** 10.3390/jimaging12080395

**Published:** 2026-08-20

**Authors:** Zhichao Luo, Weibin Luo, Peng Wang, Yuan Gu, Peimin Zhu

**Affiliations:** 1 Guangxi Nanbin Highway Construction and Development Co., Ltd., Nanning 530499, China; postluo@hotmail.com; 2 School of Geophysics and Geomatics, China University of Geosciences (Wuhan), Wuhan 430074, China; weibinluo@cug.edu.cn (W.L.); wangpengg@cug.edu.cn (P.W.); 3 Guangxi Traffic Engineering Testing Co., Ltd., Nanning 530299, China; 4 Guangzhou Marine Geological Survey, Guangzhou 511458, China; guy15@163.com

**Keywords:** pile foundation, in-service bridge, defects and damage, parallel seismic test, elastic reverse time migration

## Abstract

The conventional parallel seismic test (PST) is a widely used and highly reliable method for determining lengths of in-service bridge pile foundations. However, it relies solely on the manual interpretation of first-arrival in seismic records and often fails to detect, characterize, or geometrically define internal defects within the piles. To overcome these limitations and enable the intuitive identification of internal defects and damage within piles, this study introduces an elastic reverse time migration (ERTM) imaging algorithm based on the spectral element method, achieving high-resolution imaging of existing bridge pile foundations and their defects and damage. To suppress crosstalk between P- and S-waves during the ERTM process, a wavefield decoupling method is employed to separate the elastic wavefield into P- and S-wave components for independent imaging. Two-dimensional numerical testing on a bridge pile model with a necking defect demonstrates that ERTM can effectively image both the pile geometry and its defects, significantly improving the capability of defect detection and characterization. This approach provides more intuitive visualization for assessing the structural integrity of in-service bridge pile foundations.

## 1. Introduction

Pile foundations are the supporting structures of bridges, and their structural integrity is directly related to the safe operation of bridges. During long-term service, pile foundations not only bear regular traffic loads, but also inevitably face overload transportation, vehicle or vessel impacts, riverbed scouring, and the effects of geological disasters such as earthquakes, floods, landslides, and debris flows. As a result, structural damage such as cracks and fractures can easily occur [1,2]. In addition, for the renovation and reconstruction of some old bridges, as well as post-disaster strengthening, design and construction documents are often lacking. On-site measurement of pile length is therefore required to evaluate the bearing capacity of pile foundations. Methods commonly used to detect pile length and to identify hidden hazards such as construction-stage defects and service-stage damage in bridge pile foundations include the parallel seismic test (PST), cross-hole elastic tomography, borehole magnetic surveys [3], borehole radar, and electromagnetic induction. Compared with other approaches, the PST has been widely used in pile foundation testing because of its reliability in determining pile length and pile defects and damage.

The earliest literature on the PST dates back to Stain’s work in 1982 [4]. The PST was specifically developed in the early 1980s by the French Centre Expérimental de Recherches et d’Études du Bâtiment et des Travaux Publics (CEBTP), headquartered in Paris, to address the challenges of assessing unknown underground bridge foundations. It emerged as a borehole-based non-destructive testing (NDT) method at a time when pile integrity testing was gaining importance due to frequent construction accidents in the 1960s and 1970s. These accidents highlighted the need for reliable post-installation assessment methods beyond visual inspection or load testing. Based on the principles of seismic wave propagation, the PST was initially applied to bridge substructures with embedded pile caps or large components.

Subsequently, the PST underwent extensive research in the United States, gaining broader recognition—particularly in the mid-1990s—through the U.S. National Cooperative Highway Research Program (NCHRP). In 1995, the NCHRP Project 21-5 (“Determination of Unknown Subsurface Bridge Foundations”), led by Olson Engineering, Inc., tested the method at seven bridge sites across Colorado, Texas, and Alabama [5,6]. The PST is regarded as a versatile borehole-based NDT technique suitable for various substructure types and geological conditions, capable of successfully detecting piles beneath pile caps. Thanks to the efforts of numerous scientists and engineers, the PST has become a standard technique for detecting in-service pile foundations [7,8,9] and has found widespread application [10].

Since its inception, the core technology of the PST has remained largely unchanged. Following the year 2000, the introduction of numerical modeling, simulation, and improved interpretation algorithms addressed factors such as borehole inclination, soil stratification, and the pile-to-borehole distance (typically maintained within 1~2 m to ensure accuracy), thereby enabling the method’s application in more complex geological conditions. The PST method has been incorporated into standards such as the French AFNOR NF P94-160-3 [11] and guidelines for foundation assessment by organizations like ASTM International; its scope of application has expanded beyond bridges to include general building structures, sheet piles, and defect detection. Today, the PST method remains the most widely used non-destructive testing technique for determining the depth of unknown foundations—suitable for both metallic and non-metallic piles—without the need for calibration.

The PST method detects the internal structures, fractures, cracks, and other defects or damage within pile foundations based on the difference in the propagation velocity of transmitted elastic waves between the pile foundation and the surrounding soil. Prior to measurement, a borehole of sufficient depth must be drilled adjacent to the pile being tested. Typically, a borehole parallel to the pile foundation can be drilled at a distance of approximately 1 m from the pile, and the borehole depth needs to exceed the expected pile length by 8 to 10 m. A PVC casing (approximately 5 cm in diameter and fitted with an end cap) is commonly inserted into the borehole, which is then filled with clean water to enhance acoustic coupling for the hydrophone. During the actual testing process, the bridge pier is struck with a hammer to generate a downward-propagating seismic wave. As the seismic wave propagates downward through the pile, a portion of the wave energy is transmitted into the surrounding soil. These seismic waves are recorded by a hydrophone installed within the borehole. The hydrophone is moved vertically at set intervals, with measurements recorded at each depth to ultimately generate a depth profile of seismic data.

The pile toe can be identified by the significant increase in travel time resulting from the fact that the velocity in the surrounding soil is much lower than that in the concrete, thereby determining the pile length. Similarly, the defects and their depth can be determined by identifying subtle offsets or disruptions of the seismic first arrivals caused by velocity contrasts between the concrete and the material filling fractures or cracks (such as soil, water, or air). When the pile shaft is continuous and intact, seismic waveforms across adjacent traces exhibit high similarity and continuity; however, if low-velocity defects—such as fractures, necking, or significant mud inclusions—are present, seismic wave propagation is subject to attenuation and time delays, resulting in energy fluctuations and waveform discontinuities in the transmitted wave events recorded by adjacent receivers. An advantage of the PST is that seismic wave propagation through the soil provides information regarding the condition (e.g., stiffness) of the soil surrounding the foundation.

However, in practical applications, the PST method often suffers from the following limitations: (1) Sensitivity to geological conditions: The soil surrounding the pile is usually a non-uniform layered medium or backfill soil. The velocities and attenuations of seismic waves in such a medium change greatly, resulting in rapid changes in seismic waveforms and first-arrival events, easily causing wrong interpretation, which directly affects the detection effect [6]. (2) Theoretical errors: When determining the pile bottom depth or defect location, simplified calculation models based on ray theory introduce theoretical errors under specific pile/soil velocity ratios, leading to a certain degree of uncertainty in the results. (3) Limitations of borehole inclination: The test borehole must maintain high verticality. If the borehole tilts at depth, the actual distance between the measuring points and the pile will change, creating false velocity anomalies or depth deviations. (4) Difficulty in resolving small defects and damage: Cracks and fractures smaller than a quarter of the seismic wavelength are difficult to detect. The existence of these problems directly limits the performance of the PST method.

To overcome these limitations and accurately characterize pile defects and damage, this study introduces the elastic reverse time migration (ERTM) method to reconstruct the internal structure of pile foundations. The reverse time migration was first proposed by [12]. Its basic principle is to apply a cross-correlation imaging condition in the spatial domain between the forward-propagated wavefield excited by the sources and the backward-propagated wavefield generated from the receivers, thereby extracting high-fidelity reflectivity information at subsurface interfaces.

If the full wavefield is directly cross-correlated during ERTM, crosstalk between different wave modes, namely P- and S-waves, will generate migration artifacts and false coherent events. Therefore, accurate separation of P- and S-wave wavefields and the independent application of imaging conditions are essential for improving imaging quality. In this study, a newly developed decoupled elastic wave equation method based on the spectral element method is employed to achieve P- and S-wavefield separation [13]. This study tests a two-dimensional elastic pile foundation model containing a typical defect, validating the imaging efficacy of reverse time migration.

## 2. Methodology

### 2.1. Elastic Reverse Time Migration Method

The fundamental method for elastic reverse time migration was proposed by Sun and McMechan (1986) [14]. Assuming that $U_{s}(x,t)$ and $U_{r}(x,t)$ denote the source wavefield (forward-propagated wavefield) and the receiver wavefield (backward-propagated wavefield) at position x and time t, respectively, the standard cross-correlation imaging condition formula after considering spherical divergence of elastic seismic waves is expressed as follows:

(1) $$R(x)=\frac{\int_{0}^{\infty }U_{r}(x,t)\cdot U_{s}(x,t)dt}{\int_{0}^{\infty }U_{s}{\left(x,t\right)}^{2}dt}$$

where R(x) represents the reflection coefficient related to the changes in physical properties (density and elastic modulus) on both sides of the interface; $U_{s}(x,t)$ and $U_{r}(x,t)$ represent the particle displacement, velocity, or acceleration vector wavefields, respectively.

To satisfy the calculation of the forward- and backward-propagated wavefields, this paper utilizes the spectral element method based on the Lagrange interpolating basis functions as the fundamental computational approach for the numerical solution of the elastic wave equation [15,16].

### 2.2. Wavefield Separation

In elastic reverse time migration, if the cross-correlation imaging condition is directly applied to the total seismic wavefields, the mixing of P- and S-waves creates crosstalk, leading to migration artifacts. Therefore, separating the P- and S-wavefields is crucial. Assuming that seismic waves propagate in an isotropic elastic medium, the displacement wave equation describing the coupled motion of P- and S-waves can be expressed as follows [17]:

(2) $$\left\{\begin{array}{l} \rho \ddot{u}=(\lambda +2\mu )\nabla (\nabla \cdot u)-\mu \nabla \times \nabla \times u+f\\ \rho {\ddot{u}}^{P}=(\lambda +2\mu )\nabla (\nabla \cdot u)\\ u^{S}=u-u^{P} \end{array}\right.\mathrm{in}\;\Omega \times [0,T]$$

where ρ represents the density, and the parameters λ and μ are Lamé constants; u=u(x,t) denotes the particle displacement vector at space x∈Ω and time t∈[0,T]. In the above formula, $u=u^{P}+u^{S}$, $\ddot{u}=\partial^{2}u/\partial t^{2}$, where $u^{P}$ and $u^{S}$ correspond to the decomposed P- and S-wave displacement wavefields, respectively, and f represents the external body force vector.

Applying the spectral element method, Equation (2) can be transformed into the following two systems of second-order linear ordinary differential equations and a simple equation for calculating the S-wavefield:

(3) $$M\ddot{U}+KU=F$$

(4) $$M{\ddot{U}}^{P}+K^{P}U=0$$

(5) $$U^{S}=U-U^{P}$$

where U denotes the global displacement vector, M the global mass matrix, and K the global stiffness matrix. F represents the source external force vector. $U^{P}$ and $U^{S}$ represent the decoupled global displacement vectors for P- and S-waves, respectively; $K^{P}$ represents the global stiffness matrix associated with the P-wavefield $U^{P}$. By jointly solving Equations (3)–(5), the decomposed P-wave field $U^{P}$ and S-wave field $U^{S}$ can be obtained.

According to Equation (1), by respectively combining the separated forward-propagated P- and S-wavefields with the backward-propagated P- and S-wavefields, four imaging results can be obtained, specifically: $R_{PP}(x)$, $R_{PS}(x)$, $R_{SP}(x)$ and $R_{SS}(x)$. The four image components are used jointly to evaluate the geometry, length, integrity, and internal defects of the pile foundation. This multi-component imaging strategy helps reduce the ambiguity caused by wave-mode crosstalk and improves the reliability of defect characterization.

## 3. Numerical Imaging Experiment

### 3.1. Pile Foundation Model and Synthetic Data Analysis

**Figure 1:**
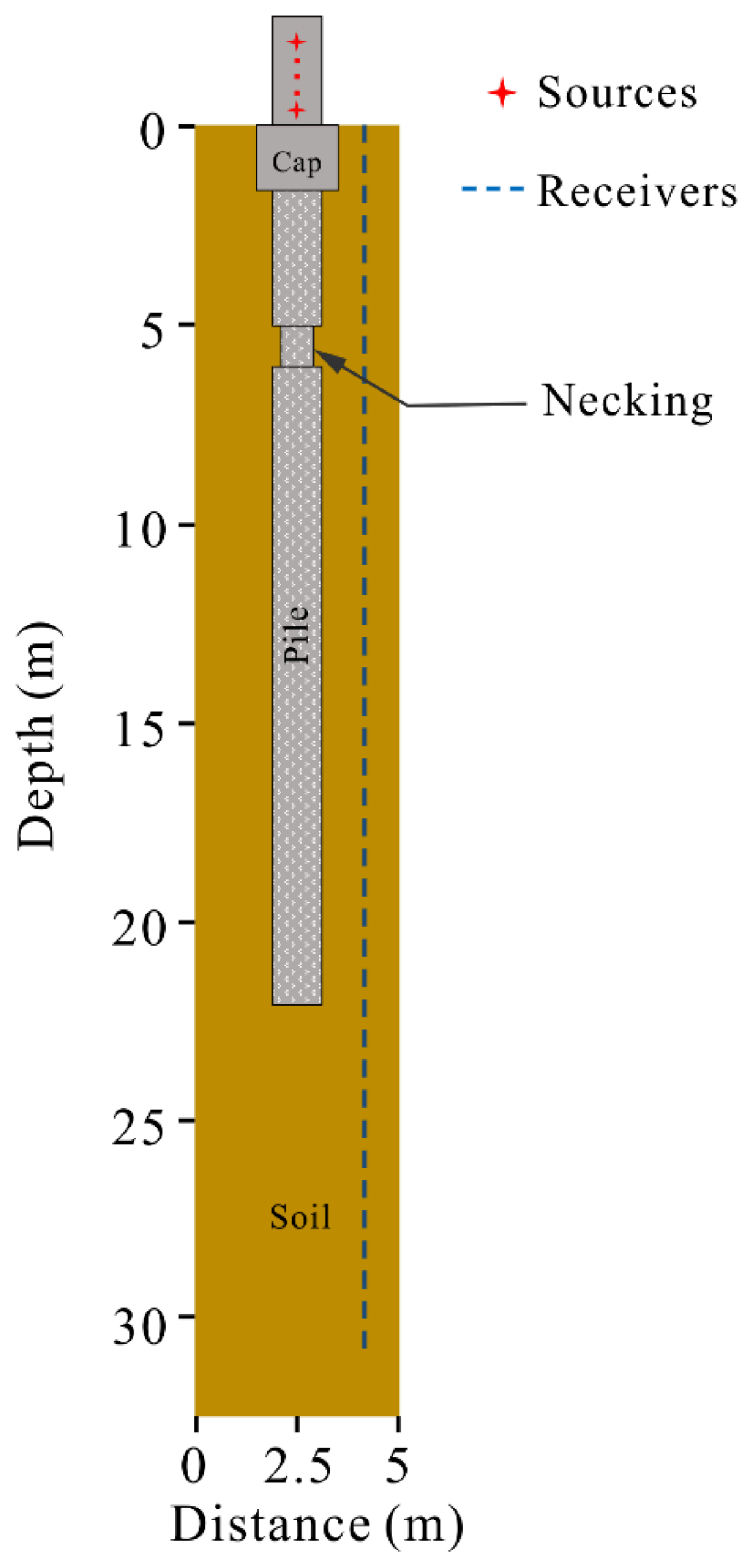
A two-dimensional model of bridge pile foundation and acquisition geometry.

To demonstrate the effectiveness of ERTM imaging for existing bridge pile foundations using parallel seismic data, this study constructed a simplified two-dimensional pile foundation model based on an actual engineering case from Qingling Interchange in Wuhan, China, as shown in Figure 1. The cylindrical pile has a diameter of 1.2 m and a length of 20 m and is cast with C25 concrete. It is connected at the top to a pile cap with dimensions of 2 m × 1.6 m. Both the pile cap and the upper pier column are cast with C30 concrete. In the model, the surrounding soil is represented as the background medium, with the ground surface defined at a depth of 0 m. In addition, a necking defect with a diameter of 0.8 m is set at a depth of 5 to 6 m along the pile body. Table 1 details the physical properties of the materials composing each part of the model. C25 and C30 refer to concrete strength grades defined in the Chinese National Standard GB50010-2010, Code for Design of Concrete Structures, (2015 Edition) [18].

**Figure 2:**
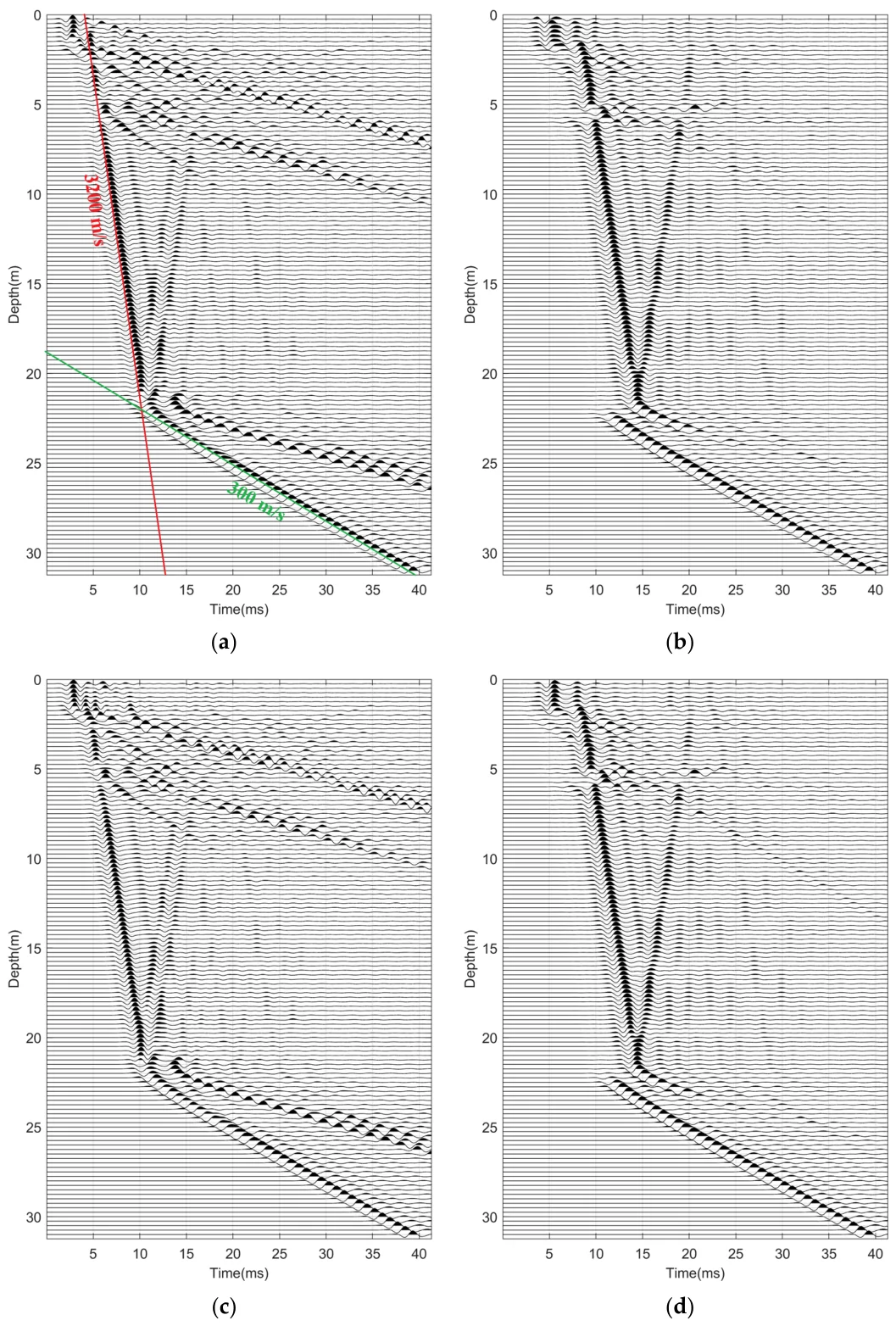
Synthetic elastic seismic data acquired with PST configuration. (**a**,**b**) X- and Z-components of the No. 1 shot; (**c**,**d**) X- and Z-components of the No. 5 shot. The slopes of the red and green lines represent the velocities in the pile material and the soil, respectively.

As shown in Figure 1, the blue dashed line indicates the position of the borehole where receivers are deployed, which is located 1 m away from the pile foundation. The upper red four-pointed stars indicate the locations of the seismic sources; a total of 124 receivers are arranged inside the borehole with a receiver spacing of 0.25 m, and the shallowest receiver is located at a depth of 0.25 m below the ground surface. Based on numerical simulation of the elastic wave equation using the spectral element method, 9 shots of elastic seismic wave records were obtained, with each shot containing 2 × 124 traces of X- and Z-component seismic data (Figure 2). In computing the synthetic seismic wave data, the model was discretized into rectangular elements of 0.1 m × 0.1 m, and seismic sources were applied as a vertical downward central force using a Ricker wavelet with a dominant frequency of 450 Hz.

Additionally, in elastic wavefield simulation and subsequent ERTM processing, the perfectly matched layer (PML) technique was implemented to suppress artificial reflections and simulate an infinite domain. According to the Courant–Friedrichs–Lewy (CFL) stability condition [19], the time steps for both the elastic wavefield simulation and ERTM were identically set to 0.002 ms. The total recording duration of synthetic seismic data was set to 45 ms.

The sources for shots 1 to 9 were excited at positions 0.2 to 1.8 m at an interval of 0.2 m above the tie beam (pile cap), respectively. Figure 2 shows only the elastic seismic data for the 1st and 5th shots.

From the two auxiliary lines drawn on Figure 2a, the pile foundation (red) and the soil (green) can be clearly distinguished via their velocities being approximately 3520 m/s and 300 m/s, respectively. The intersection of the two lines indicates the pile toe position. Additionally, at a depth of 5 to 6 m, a break of the seismic event can be observed due to the presence of the necking defect, where the first-arrival time is delayed. This is mainly due to the additional 0.2 m propagation distance through the soil of lower velocity. These results only provide us with information about the pile length and the defect location but make it difficult to determine the geometric shape of the defect and the overall morphology of the pile foundation. Especially when defects and damage are smaller and the surrounding soil is heterogeneous, small defects and damage are difficult to distinguish.

### 3.2. Imaging Results

**Figure 3:**
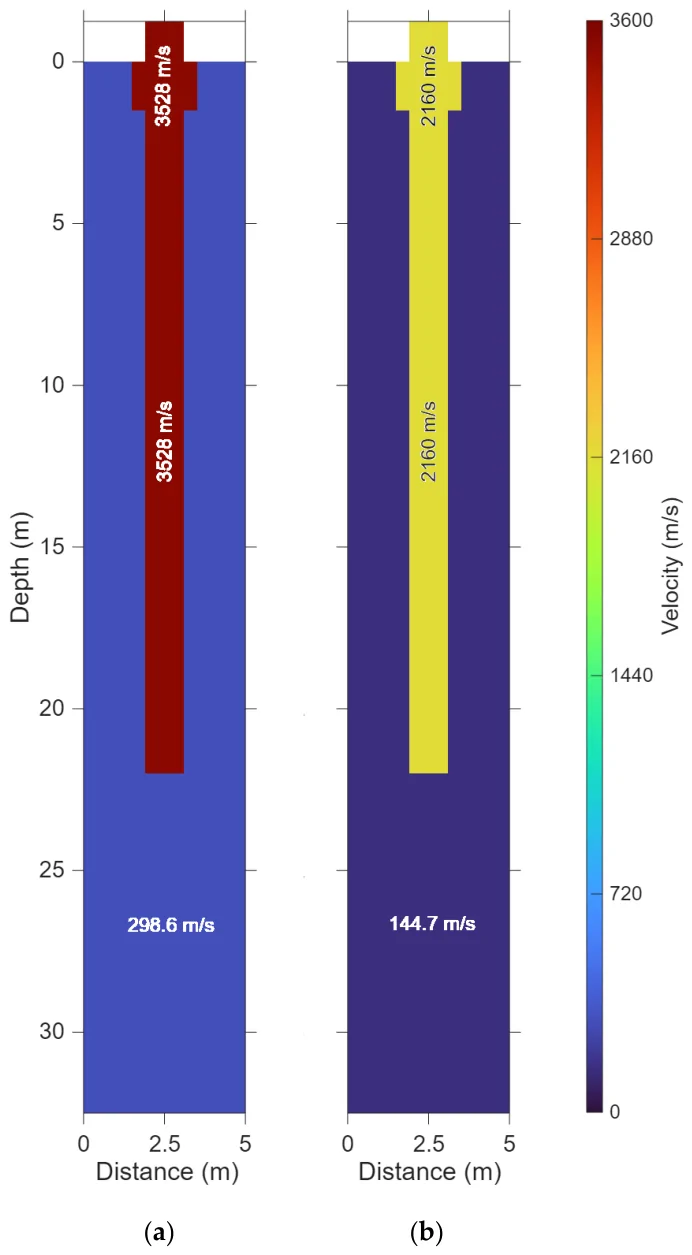
Velocity models for imaging by ERTM. (**a**,**b**) are P- and S-wave velocity, respectively.

**Figure 4:**
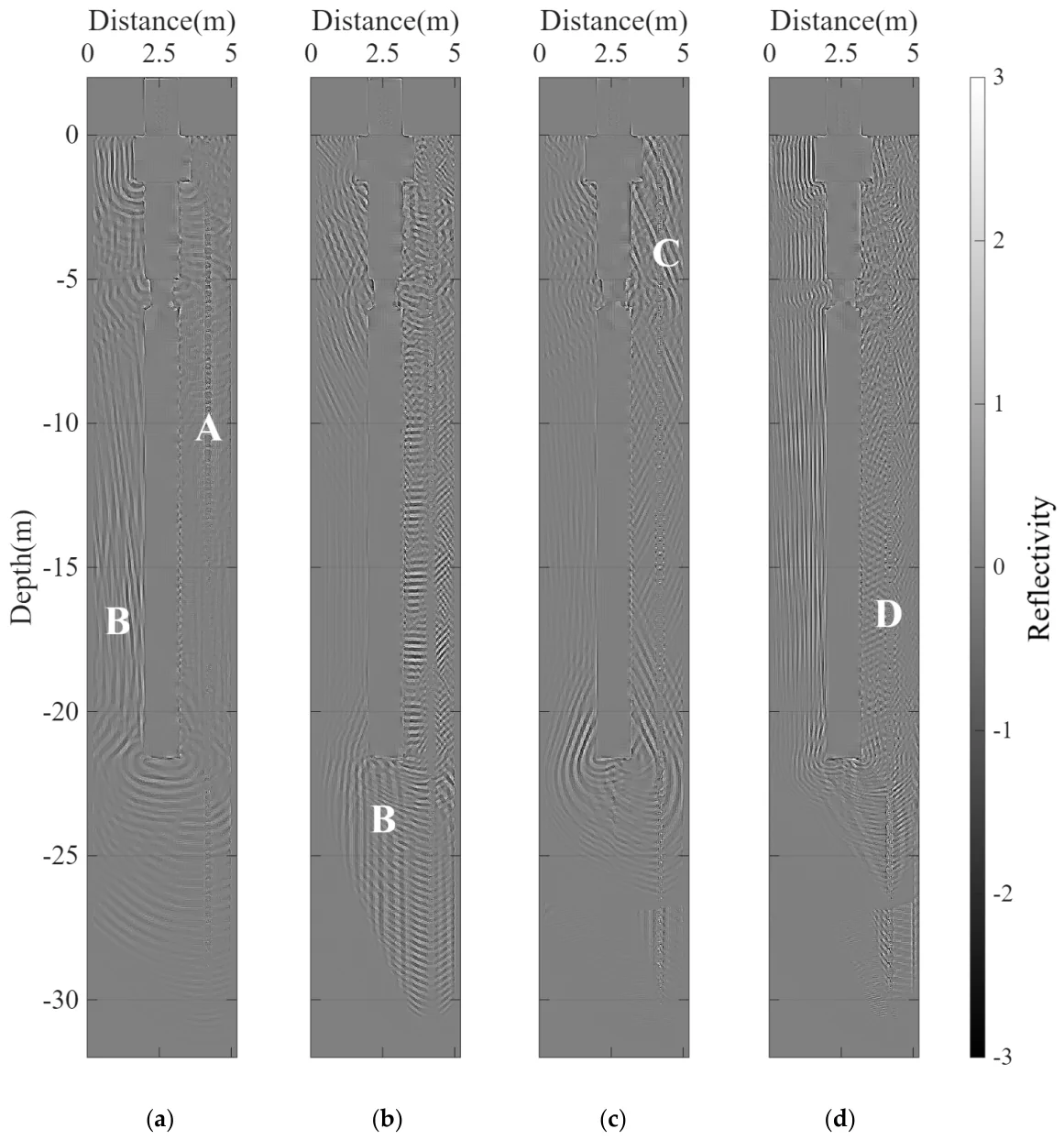
Imaging results using ERTM with all 9 shots of data. (**a**–**d**) are the migration results for PP-, PS-, SP- and SS-waves, respectively. The characters A denotes the dot-like artifacts, B and C denote the false coherent events, and D represents the undulating background noise.

In this study, the imaging process of ERTM is essentially the same as that in petroleum seismology. First, we established a migration velocity model. The construction of the velocity model was based on the structural design drawings of the bridge pile foundation, prior geotechnical investigations of the surrounding soil, and the velocity analysis process shown in Figure 2a. To approximate real-world scenarios and avoid “inverse crime”, our migration velocity model was designed as a defect-free velocity model (Figure 3). Figure 4 displays the migration results for all nine shots of data.

The ERTM imaging computations were performed on a DELL server manufactured in USA equipped with an Intel Xeon CPU, 128 GB of RAM, and an NVIDIA A100 GPU (80 GB VRAM). Generating the image for a seismic dataset comprising nine shot gathers required approximately 1.5 h of processing time.

The imaging results show that the elastic reverse time migration method can clearly reconstruct the geometry of the pile body and successfully locate the necking defect. As can be seen from the imaging results of the four different wavefield modes in the figure, the overall geometry of the pile cap and pile foundation, as well as the geometry of the defect, are basically identical to the actual true model shown in Figure 1, demonstrating the effectiveness of ERTM. Furthermore, the imaging results inside the pile foundation are very clean, and the boundaries of the pile foundation and the necking defect can be perfectly delineated. In particular, both sides of the necking defect with a lateral length of 0.2 m are clearly visible, indicating that the imaging resolution of ERTM can achieve an accuracy of at least 0.2 m.

It is worth noting that there are various prominent noises outside the pile foundation and pile cap. These noises mainly include: dot-like artifacts, false coherent events, and undulating background noise labeled as A, B, C, and D in Figure 4. A represents dot-like strong energy artifacts caused by the source and receivers. These artifacts usually manifest as intense high-amplitude background noise, the root cause of which is the cross-correlation results of high-energy transmitted direct waves. The false coherent events labeled as B and C are spurious coherent events generated due to the inherent limitations of standard imaging conditions, strong velocity contrasts, and multi-pathed wavefields propagating in the model (such as internal multiples). Research communities have conducted extensive studies on suppression methods for such artifacts [20,21]. However, these existing suppression methods are not applicable to the transmission-type observation configuration used in this study (Figure 1). The undulating background noise indicated in Figure 4 is primarily caused by the specific acquisition geometry of the target investigated in this paper. In a normal acquisition geometry for reverse time migration, if the recording aperture is large enough, elastic waves observed from multiple different azimuths (directions) can eliminate most of the undulating background noise in the imaging results through multi-shot stacking [22]. The aperture mentioned here means that the receiver array is sufficiently long. In this study, relative to the length of the pile body, the aperture corresponding to the receiver array is already large enough, but the azimuth is too narrow, consisting only of elastic waves propagating downward from the source along the pile body and transmitting into the soil. Therefore, for the PST observation scenario of in-service bridge pile foundations, researching a novel ERTM noise suppression method is highly necessary.

Moreover, although undulating background noise and false coherent events exist across all four modes (PP, PS, SP, and SS) of the ERTM imaging results, they differ from one another and possess distinct characteristics. Analyzing and comparing these results together helps distinguish the pile foundation and defects from background noise and coherent noise (false coherent events).

**Figure 5:**
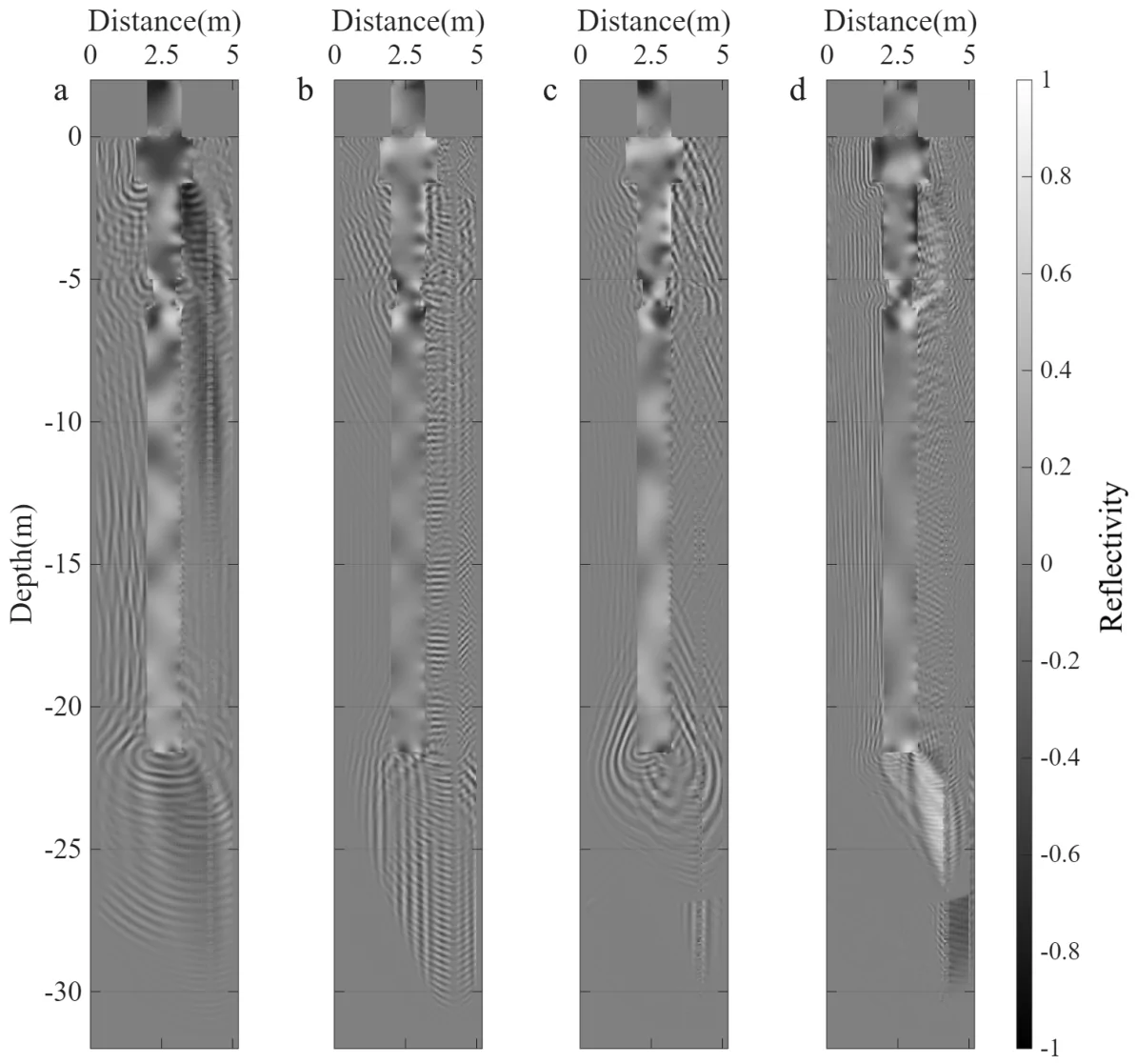
Imaging results using ERTM with only No. 1 shot data. (**a**–**d**) are the migration results for PP-, PS-, SP- and SS-waves, respectively.

Under normal circumstances, the PST method uses only a single shot of data to analyze the length and defects of the pile foundation. Figure 5 shows the migration results using only the first shot of data. Although the boundaries of the pile foundation, pile cap, and defect in Figure 5 are still relatively clear, compared to the joint migration results of nine shots (Figure 4), the imaging quality is relatively degraded, and low-wavenumber noise is present inside the pile foundation. Therefore, in practical work, multi-shot observations should be conducted as much as possible to improve imaging quality.

Notably, the imaging results (Figure 4) clearly reveal a necking defect. On both the left and right sides of the pile, a recessed notch, measuring 1.01 m in length and 0.2 m in width, is clearly visible. According to the Fresnel zone theory, geological features with dimensions smaller than a quarter wavelength (λ/4) generally fall below conventional resolution limits and are difficult to resolve. In this study, the elastic wavelengths within the concrete pile and surrounding soil are 7.84 m and 0.67 m, respectively, corresponding to quarter wavelengths of 1.96 m and 0.17 m. However, this study successfully outlined the complete boundaries of the necking defect with a diameter of 0.8 m and a length of 1 m, indicating that the ERTM method possesses high-resolution imaging capabilities when utilizing parallel seismic wave data for pile foundation testing.

We carefully compared the differences between the imaging results and the true model, including the estimation errors for pile toe depth, defect depth, and defect size. These results are summarized in Table 2, and the maximum relative error is less than 2%, which is attributed to the noise-free and idealized nature of the synthetic numerical experiment.

It is worth noting that the migration velocity model (Figure 3) used in our numerical experiment departs from the exact true model. Specifically, the migration velocities deviate from the true design values by 1.5% (P-wave) and 1.53% (S-wave) at the pile cap and pier and by 91.5% (P-wave) and 93.3% (S-wave) at the necking defect. The successful imaging results demonstrate that the ERTM method possesses a notable tolerance to velocity mismatch.

## 4. Discussion and Conclusions

1. A parallel seismic test method based on ERTM for in-service bridge pile foundations is presented; a 2D numerical test demonstrates that the geometric profiles of the pile foundation and its defects can be reconstructed with high accuracy.
2. Among the four components of the ERTM imaging results, PP, PS, SP, and SS, the background noise and false coherent events are different. Nevertheless, all four components can accurately delineate the geometry of the pile foundation and the defect. Therefore, comparing the imaging results of the four components to determine the pile length and integrity, as well as the geometry and nature of the defect, is a reliable and feasible approach.
3. In the four components of the ERTM imaging results, namely PP, PS, SP, and SS, undulating background noise and false coherent events are relatively significant. These noises are mainly caused by two factors: first, false coherent events generated by the inherent imaging condition of reverse time migration; and second, undulating background noise caused by insufficient aperture and azimuth coverage of the observed data. Therefore, it is necessary to study advanced noise suppression methods for ERTM imaging under the parallel seismic observation configuration.
4. To further suppress the background noises and false coherent events for PST layouts, we will expand our research to mitigation strategies that include directional Poynting-vector-based back-scattering suppression, angle-domain common image gathers (ADCIGs), and multi-component weighted stacking (combining PP and PS imaging results destructively against coherent events).
5. Compared with the currently used parallel seismic method, the ERTM-based method proposed in this study for imaging the internal structure and defects of pile foundations requires: first, three-component sensors to acquire elastic vector wavefields; and second, an increased number of shots to suppress imaging noise and improve imaging quality.
6. Although multi-shot excitation and three-component sensors increase data acquisition requirements compared to traditional single-shot PST, the operational overhead remains well within practical limits for civil engineering testing. Specifically, for a 30 m borehole requiring 120 depth positions with a 0.25 m spacing using a string of six three-component geophones, nine shots involve a total of 180 hammer strikes. Because the dominant time-consuming and costly component of PST is drilling and casing the borehole—while seismic recording per hammer strike takes only a few tens of milliseconds—performing multiple surface hammer strikes at predetermined pier locations adds negligible time and labor overhead. When weighed against the significant diagnostic benefits of transforming PST from a qualitative curve-fitting method into a high-resolution spatial imaging tool capable of resolving fine geometric details, this multi-shot ERTM scheme offers a highly favorable balance between field feasibility and investigative precision.
7. To further verify the method’s robustness, in future work, we will investigate 3D models and more complex damage patterns, such as fractures, mud inclusions, and multi-defect coexistence, as well as surrounding layered soil. Unfortunately, our team currently does not have such equipment and therefore cannot verify the method using measured field data at this stage. In future research, the team will attempt to apply the method to imaging using actual data to verify its reliability and engineering application value under complex geological conditions.

## Figures and Tables

**Table 1:** Material parameters.

Materials	P-Wave Velocity (m/s)	S-Wave Velocity (m/s)	Density (kg/m^3^)
Concrete C25	3528	2160	2500
Concrete C30	3581	2193	2600
Soil	298.6	144.7	1800

**Table 2:** Comparison between dimensions of true model and imaging results.

Component & Their Positions	True Dimension (m)	Imaging Results		
		Measured Dimension (m)	Absolute Error (m)	Relative Error (%)
Pile toe depth	22.0	22.0	0.0	0.0
Pile diameter	1.2	1.21	0.01	0.08
Defect top depth	5.0	5.0	0.0	0.0
Defect length	1.0	1.01	0.01	1.0
Defect width	0.8	0.79	0.01	1.25

## Data Availability

The raw data presented in this study are available on request from the corresponding author.
